# Diagnostic contribution of anaerobic bottles in pediatric blood cultures: experience at a tertiary children’s hospital

**DOI:** 10.1128/jcm.00764-26

**Published:** 2026-09-30

**Authors:** Ju Hyeong Lee, Baek Sup Shin, Taek Soo Kim, Sue Shin, Ki Wook Yun, Eun Hwa Choi, Jae Hyeon Park

**Affiliations:** 1 Department of Laboratory Medicine, Seoul National University Hospital 58927, Seoul, South Korea; 2 Department of Laboratory Medicine, Seoul National University College of Medicine 37990, Seoul, South Korea; 3 Department of Pediatrics, Seoul National University Children's Hospital 230993, Seoul, South Korea; 4 Department of Laboratory Medicine, Seoul National University Boramae Medical Center 65633, Seoul, South Korea; 5 Department of Pediatrics, Seoul National University College of Medicine 37990, Seoul, South Korea; Children's Hospital Los Angeles, Los Angeles, California, USA

**Keywords:** blood culture, bacteremia, child, infant, *Staphylococcus aureus*, *Escherichia coli*

## Abstract

Routine use of anaerobic bottles in pediatric blood cultures remains controversial because diagnostic yield must be balanced against limited sampling volume. We retrospectively analyzed paired pediatric aerobic–anaerobic blood cultures collected from patients younger than 18 years at a tertiary children’s hospital from January 2020 through December 2024. Accession-defined paired sets received within 15 min were grouped into culture episodes; episodes containing at least two paired sets comprised the primary cohort. Among 24,485 multi-set episodes from 8,658 patients, 999 (4.1%) were positive, and 387 (1.6%) were potential-contaminant episodes. Confirmed anaerobic-bottle contribution was observed in 208 positive episodes (20.8%), comprising additional organism recovery in 159 (15.9%) and detection more than 4 h earlier in 49 (4.9%); the absolute added yield was 8.5 per 1,000 episodes. Obligate anaerobes were identified in 11 positive episodes, whereas the majority of the contribution came from facultative organisms, particularly *Staphylococcus aureus*, *Escherichia coli*, and viridans group streptococci. Contribution was highest in the emergency room (28.2%) and neonatal intensive care unit (25.0%). Compared with the general ward, adjusted odds were higher in the emergency room (adjusted odds ratio, 1.83; 95% confidence interval, 1.23 to 2.73) and lower in the hemato-oncology ward (0.43; 0.21 to 0.85). Contribution was 21.8% among children younger than 4 years. The overall median within-accession time-to-positivity difference (anaerobic minus Peds Plus/F) was small (−0.17 h), although selected organisms showed earlier anaerobic detection. Anaerobic bottles provided complementary diagnostic information in approximately one-fifth of positive pediatric blood culture episodes, mainly through additional recovery rather than uniformly earlier detection.

**IMPORTANCE:**

Children often have only a small amount of blood available for culture, making the choice of culture bottles important. In this hospital-wide study, anaerobic bottles added diagnostic information in about one-fifth of positive pediatric blood culture episodes, mainly by recovering organisms that were not found in the paired pediatric aerobic bottle. Most of this benefit involved common bloodstream pathogens that can grow with or without oxygen, including *Staphylococcus aureus* and *Escherichia coli*, rather than rare obligate anaerobes. A similar overall contribution proportion was observed among children younger than 4 years. However, the pediatric aerobic bottle recovered more organisms overall in direct paired comparisons. These findings support viewing the two bottle types as complementary and emphasize that pediatric blood culture practice should balance adequate total blood volume with thoughtful allocation between bottle types.

## INTRODUCTION

Blood culture remains the reference standard for diagnosing bloodstream infections in children and for guiding targeted antimicrobial therapy (1). In pediatric practice, however, diagnostic yield depends not only on timely sampling and microbiologic processing but also on inoculated blood volume and the number of cultures obtained, both of which are often constrained by low circulating blood volume and difficult venipuncture (2–5). Inadequate sampling can reduce pathogen recovery (2–5), and low-volume cultures are more likely to yield contaminant isolates (4). Contaminated blood cultures impose downstream burdens on children, families, and the health care system (6) and may lead to subsequent unnecessary health care utilization (7), underscoring the need for diagnostic stewardship in pediatric blood culture practice (8).

Within this stewardship framework, the role of routine anaerobic bottles in children remains controversial. Historically, pediatric practice often favored selective rather than universal anaerobic culture because obligate anaerobic bacteremia is uncommon in children and because blood culture sensitivity is highly volume dependent when only small samples can be obtained (2, 5, 9). The practical question is therefore not only whether anaerobic bottles add value but also how limited pediatric blood volume should be allocated between bottle types. In neonates, a prospective trial showed that distributing 1 mL across paired aerobic and anaerobic bottles improved recovery compared with inoculating the same volume into a single aerobic bottle, illustrating that bottle strategy and blood-volume stewardship are interdependent rather than competing issues (10).

More recent pediatric studies have challenged the older restrictive view. In a pediatric emergency department, adding anaerobic bottles increased recovery of obligate and facultative anaerobes and improved detection of clinically relevant pathogens (11). Similar observations have been reported in neonatal and pediatric intensive care settings, where anaerobic bottles identified additional pathogens and, in some subsets, flagged positive earlier than aerobic bottles alone (12–14). In a contemporary paired-bottle study that included specimens from both adults and children, routine anaerobic bottles enhanced microorganism recovery and improved time to positivity for selected organisms, supporting the continued value of anaerobic bottles beyond the detection of obligate anaerobes alone (15). Recent Korean pediatric data are consistent with this pattern: Noh et al. found that obligate anaerobes were rare but that many facultative anaerobes were recovered only from anaerobic bottles (13).

Nevertheless, most prior studies have been performed at the bottle level, have focused on selected units, such as the emergency department, neonatal intensive care unit (NICU), or pediatric intensive care unit (PICU), or have included mixed adult–pediatric populations, making it difficult to estimate the episode-level diagnostic contribution of anaerobic bottles across routine pediatric practice (11–13, 15, 16). This distinction is clinically relevant because recent stewardship interventions show that optimizing blood volume and culture strategy can change pathogen recovery in children, but hospital-wide episode-level data that integrate these issues in routine care remain limited (17, 18).

Therefore, we conducted a hospital-wide retrospective study to evaluate the diagnostic contribution of anaerobic bottles in routine pediatric blood cultures, including additional organism recovery, earlier detection, and variation by organism and clinical setting. We also assessed whether the overall findings were preserved among children younger than 4 years.

## MATERIALS AND METHODS

### Study design and patient population

This retrospective observational study included blood culture sets collected from patients younger than 18 years at Seoul National University Children’s Hospital from 1 January 2020 to 31 December 2024. At our institution, pediatric blood cultures were routinely ordered as a paired test comprising one BD BACTEC Peds Plus/F bottle and one BD BACTEC Lytic/10 Anaerobic/F bottle under the same laboratory accession number; the anaerobic bottle was not a separately selected add-on. A bottle was defined as an individual culture vial, and a paired blood culture set was defined as the Peds Plus/F–anaerobic bottle pair sharing one accession. Bottle pairing was based solely on the shared laboratory accession number and therefore did not require a separate time criterion. Submissions containing only one of the two required bottles were routinely recorded in the laboratory inappropriate-specimen log; review of this log identified no incomplete paired submissions in the study cohort. Multiple paired sets from the same patient were grouped into an operational culture episode using an anchor-based 15-min laboratory-receipt window; all paired sets assigned to an episode were received within 15 min of the first paired set in that episode. Because the pediatric microbiology laboratory operated continuously 24 h per day, 7 days per week, and blood culture bottles were accessioned upon arrival without scheduled batch processing, laboratory receipt time was the most reliable routinely recorded marker of contemporaneous submission.

**Fig 1:**
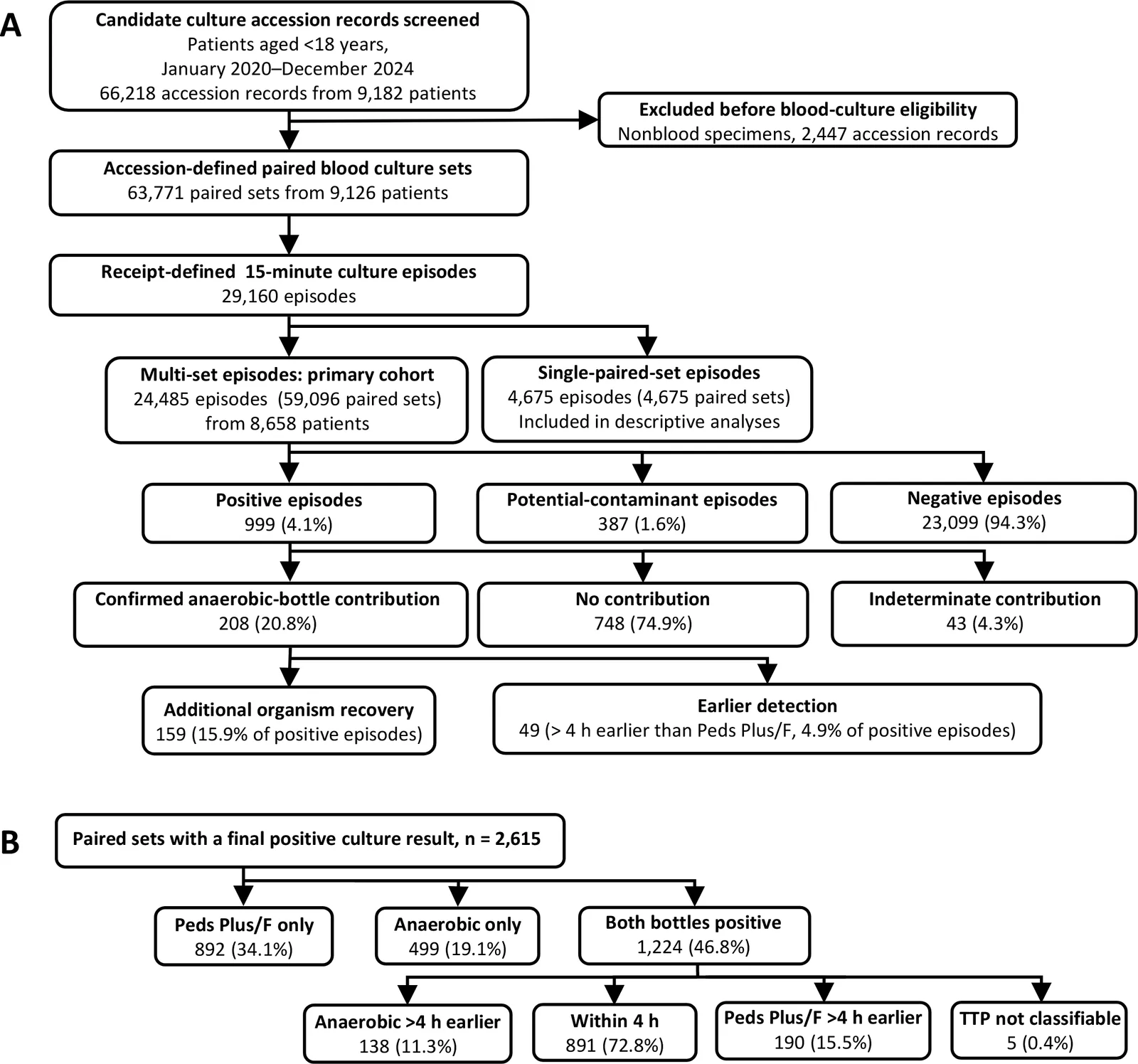
Study cohort construction and within-accession paired-bottle recovery patterns. (**A**) An accession-defined paired set comprised one Peds Plus/F bottle and one Lytic/10 Anaerobic/F bottle under the same laboratory accession. Paired sets received within 15 min of the first set were grouped into a receipt-defined culture episode. Episodes containing one paired set were included in descriptive analyses, whereas episodes containing ≥2 paired sets comprised the primary cohort; polymicrobial episodes were included. Percentages for episode outcomes and contribution categories were calculated among the 24,485 primary episodes and 999 positive episodes, respectively. (**B**) Within-accession recovery patterns among 2,615 classifiable paired sets with a final positive culture result. Timing categories and their percentages were calculated among the 1,224 paired sets positive in both bottles; within 4 h indicates an absolute TTP difference of ≤4 h. TTP, time to positivity.

A clinical encounter was defined by a common hospitalization, emergency department visit, or outpatient visit identifier. Encounter information was used for linkage and an outcome-independent sensitivity analysis but did not define the primary analytic unit. The primary analysis included culture episodes containing at least two independently accessioned paired sets, which allowed common-commensal recovery to be assessed across sets. Episodes containing a single paired set were excluded from the primary cohort and included only in descriptive analyses because common-commensal recovery could not be adjudicated by the repeated-set criterion. All eligible multi-set episodes were included without outcome-based representative selection, and patients could contribute more than one episode. Patient clustering was accounted for in the multivariable logistic regression and Cox models using patient-clustered robust standard errors. Nonblood specimens were excluded, whereas polymicrobial episodes were included and evaluated at the organism level. The study selection process is shown in Fig. 1A. This study was reported in accordance with the Strengthening the Reporting of Observational Studies in Epidemiology (STROBE) guideline for observational studies (19).

### Blood culture sample collection and processing

Blood cultures were processed using the BD BACTEC FX system (Becton Dickinson, Sparks, MD, USA). Blood samples were collected using a standardized aseptic technique in accordance with institutional practice (20). Because blood volume is a major determinant of pediatric blood culture yield, the institutional weight-based protocol recommended total blood culture volumes ranging from 2 mL in patients weighing ≤1.0 kg to 40–60 mL in those weighing >36.3 kg, with intermediate targets of 4, 6, and 20 mL for patients weighing 1.1–2.0, 2.1–12.7, and 12.8–36.3 kg, respectively. The volume was allocated across one or two paired sets according to weight. One paired set was recommended for patients weighing ≤1.0 kg and two paired sets for those weighing >1.0 kg. BD BACTEC Peds Plus/F and BD BACTEC Lytic/10 Anaerobic/F culture vials (Becton Dickinson, Sparks, MD, USA) were used for aerobic and anaerobic culture, respectively; hereafter, these are referred to as the Peds Plus/F and anaerobic bottles. Within each paired set, the protocol specified equal division of the available blood between the two bottles, even when the obtained volume was small. No body-weight threshold triggered a transition to a conventional adult aerobic bottle because only this paired-bottle regimen was supplied for pediatric blood cultures in the children’s hospital. Bottle-level inoculated volumes were not recorded; therefore, adherence to the recommended volumes and allocation could not be assessed. All bottles were incubated at 35°C and continuously monitored for up to 120 h or until a positive signal was detected. Instrument-derived time to positivity (TTP) was defined as the interval from bottle loading to the positive signal. Positive bottles underwent Gram staining and subculture on appropriate aerobic and anaerobic media. Final organism recovery was determined from the laboratory information system; instrument signals without final organism recovery were not counted as positive culture events. Organisms were identified according to the routine clinical microbiology workflow in use at the time of culture positivity, including conventional biochemical methods and automated identification systems, such as VITEK 2, MicroScan WalkAway 96 plus, and BD Phoenix M50; matrix-assisted laser desorption/ionization time-of-flight mass spectrometry (MALDI-TOF MS; Bruker Biotyper, Bruker Daltonics, Bremen, Germany) was introduced later in the study period.

### Data collection and definitions

Patient identifier, age, sex, laboratory receipt time, sampling location, specimen source, final culture result, organism identification, bottle-specific positive-signal information, and instrument-derived TTP were extracted from the laboratory and clinical information systems. Age was categorized as <1, 1–4, 5–9, 10–14, and 15–17 years for descriptive analyses and as <1, 1–4, and 5–17 years for the primary regression analysis. A separate subgroup analysis was conducted among children aged <4 years, and age was dichotomized as <4 versus 4–17 years in the exploratory Firth analysis. Sampling location was categorized as hemato-oncology ward, neonatal intensive care unit (NICU), pediatric intensive care unit (PICU), pediatric emergency room, and general ward. The collection source for each paired blood culture set was recorded as peripheral blood, central-catheter blood, or blood from a Hickman catheter lumen. Central-catheter and Hickman-lumen samples were grouped as intravascular-line blood for analysis. Because the two bottles within a paired set shared the same laboratory accession, they also had the same recorded collection source. Episode-level source composition was classified as peripheral only, line only, or mixed peripheral and line.

Typical common commensals were prespecified using a CLSI M47-informed organism list (21). The same common commensal recovered from two or more independently accessioned paired sets within the same episode was classified as a pathogen under the repeated-set criterion. In contrast, a common commensal recovered from only one paired set within a multi-set episode was classified as a potential contaminant. An episode was classified as positive if at least one pathogen was identified, as a potential-contaminant episode if no pathogen but at least one potential contaminant was identified, and as negative if all paired sets had final no-growth results. Common-commensal recovery in single-paired-set episodes was reported descriptively and was not adjudicated as contamination. A positive episode containing more than one pathogen was classified as polymicrobial. Polymicrobial episodes were included; organism-specific contribution was assessed when bottle attribution was possible, and unresolved attribution was classified as indeterminate. Obligate anaerobes were identified using a prespecified curated species list.

Anaerobic-bottle diagnostic contribution was assessed separately for each pathogen within an episode. Additional recovery was defined as recovery of the pathogen from at least one anaerobic bottle with no recovery of the same pathogen from any Peds Plus/F bottle in the episode. Earlier detection was defined when the earliest anaerobic-bottle TTP was more than 4 h shorter than the earliest Peds Plus/F-bottle TTP for the same pathogen (14). The two earliest values could arise from different paired sets within the episode; this measure was therefore interpreted as episode-level earlier detection rather than a direct paired-bottle TTP comparison. An episode was classified as having confirmed anaerobic-bottle contribution if at least one pathogen met either criterion and as having no contribution when all pathogens were evaluable and none met either criterion. A positive episode without confirmed contribution was classified as indeterminate when bottle-specific organism attribution or TTP assessment was incomplete. In supplementary within-accession analyses, recovery was classified as Peds Plus/F only, anaerobic only, both bottles, or neither bottle. Direct paired TTP comparisons were restricted to monomicrobial paired sets in which the same organism was recovered from both bottles and both TTP values were valid. Bottle-specific inoculated volumes were unavailable, and equal volumes were not assumed.

### Statistical analysis

Statistical analyses were performed using R software (version 4.5.3; R Foundation for Statistical Computing, Vienna, Austria). Because this retrospective study included all eligible blood culture records during the study period, no formal sample size calculation was performed. Continuous variables were summarized as medians with interquartile ranges, and categorical variables as counts and percentages. The primary descriptive analysis was conducted among receipt-defined episodes containing at least two paired sets. The primary contribution proportion used all positive episodes as the denominator; the proportion among contribution-evaluable positive episodes was also reported. Indeterminate episodes were included in the all-positive denominator but excluded from binary regression models. Absolute added yield was calculated as episodes with confirmed contribution divided by all primary multi-set episodes and was expressed per 1,000 episodes.

Factors associated with anaerobic-bottle contribution were evaluated among contribution-evaluable positive episodes using multivariable logistic regression with patient-clustered robust standard errors to account for repeated episodes within patients. The primary model included age group, sex, sampling location, and calendar year. Adjusted odds ratios with 95% confidence intervals were reported, and global effects were evaluated using robust Wald tests. Separate sensitivity analyses (i) additionally adjusted for episode-level collection-source composition, (ii) additionally adjusted for any pediatric complex chronic condition (CCC) defined using diagnosis codes recorded during the 365 days before or on the episode date (22), and (iii) restricted the cohort to the earliest eligible multi-set episode per clinical encounter. Organism distributions according to sampling location and findings among children younger than 4 years, single-paired-set episodes with common-commensal recovery, episodes involving obligate anaerobes, and polymicrobial episodes were summarized descriptively.

In supplementary within-accession analyses, bottle-recovery patterns among paired sets with a final positive laboratory report were compared using the exact McNemar test. Matched odds ratios with 95% confidence intervals were calculated from discordant paired sets. TTP was compared using the paired Wilcoxon signed-rank test among both-positive monomicrobial paired sets. TTP difference was calculated as anaerobic-bottle TTP minus Peds Plus/F-bottle TTP; negative values indicated earlier positivity in the anaerobic bottle.

For exploratory organism-specific analyses, cumulative positivity was estimated as one minus the Kaplan–Meier survival function. Episode-level analyses compared the earliest TTP for the target organism among all Peds Plus/F bottles with the earliest TTP among all anaerobic bottles within each receipt-defined multi-set episode. Within-accession analyses directly compared the two bottles sharing the same accession and were restricted to monomicrobial paired sets. When the target organism was not recovered from a bottle type, that observation was right-censored at 120 h. Final recovery was compared using the exact McNemar test, and TTP among both-positive units was compared using the paired Wilcoxon signed-rank test. Cox models were stratified by the episode–organism unit or accession-defined paired set and used patient-clustered robust standard errors to account for repeated observations from the same patient.

Exploratory Firth-penalized logistic regression was used to evaluate factors associated with an episode-level anaerobic-bottle lead of more than 4 h among both-positive episode–organism units from monomicrobial episodes with valid paired TTP values. Covariates included age group (<4 versus 4–17 years), sampling location, and organism group. Organism-specific analyses of *Staphylococcus aureus*, *Escherichia coli*, and *Enterobacterales* were considered exploratory. All tests were two-sided, and *P* < 0.05 was considered statistically significant. No adjustment for multiple comparisons was applied to exploratory analyses.

## RESULTS

### Blood culture results

During the 5-year study period, 63,771 accession-defined paired blood culture sets from 9,126 patients were grouped into 29,160 receipt-defined 15-min culture episodes. Of these, 4,675 episodes contained a single paired set, whereas 24,485 contained at least two paired sets and comprised the primary cohort (Fig. 1A). The primary cohort included 8,658 patients. Of the 24,485 multi-set episodes, 23,099 (94.3%) were negative, 999 (4.1%) were positive, and 387 (1.6%) were classified as potential-contaminant episodes. Among the 999 positive episodes, anaerobic-bottle contribution was confirmed in 208 (20.8%), including 159 (15.9%) with additional organism recovery and 49 (4.9%) with earlier detection by more than 4 h. Forty-three positive episodes were indeterminate because bottle-specific organism attribution or TTP assessment was incomplete. The contribution rate was 21.8% (208/956) among contribution-evaluable positive episodes. The absolute added yield was 0.85% of all multi-set episodes, corresponding to 8.5 per 1,000 multi-set episodes.

### Study population characteristics

The median age at the episode date was 5.0 years (interquartile range [IQR], 1.0–11.0 years), and 57.5% of episodes occurred in male patients (Table 1). Infants younger than 1 year accounted for 22.0% of episodes and children aged 1–4 years for 27.8%. The emergency room was the most frequent sampling location (45.1%), followed by the general ward (22.3%), hemato-oncology ward (18.9%), NICU (7.6%), and PICU (6.1%). Most episodes contained two paired sets (74.5%). Across all 63,771 accession-defined paired sets, 43,684 (68.5%) were collected from peripheral blood and 20,087 (31.5%) from an intravascular line. At the episode level, 16,057 (65.6%) were peripheral only, 4,015 (16.4%) line only, and 4,413 (18.0%) mixed peripheral and line.

**TABLE 1:** Characteristics of the primary multi-set culture-episode cohort^*a*^^*,^b^*^

Characteristic	Value
Primary multi-set episodes	24,485
Unique patients	8,658
Age, median [IQR], years	5.0 [1.0–11.0]
Age group	
<1 year	5,397 (22.0%)
1–4 years	6,810 (27.8%)
5–9 years	5,217 (21.3%)
10–14 years	4,886 (20.0%)
15–17 years	2,175 (8.9%)
Sex	
Male	14,075 (57.5%)
Female	10,410 (42.5%)
Sampling location	
General ward	5,472 (22.3%)
Emergency room	11,039 (45.1%)
NICU	1,855 (7.6%)
PICU	1,487 (6.1%)
Hemato-oncology ward	4,632 (18.9%)
Paired sets per episode, median [IQR]	2 [2–3]
Paired-set count	
2 paired sets	18,235 (74.5%)
3 paired sets	3,546 (14.5%)
≥4 paired sets	2,704 (11.0%)
Collection-source composition	
Peripheral only	16,057 (65.6%)
Intravascular line only	4,015 (16.4%)
Mixed peripheral and line	4,413 (18.0%)

^*a*^ IQR, interquartile range; NICU, neonatal intensive care unit; PICU, pediatric intensive care unit.

^*b*^ Data are presented at the episode level. Individual patients could contribute more than one episode. Collection source composition was classified across all paired sets within an episode.

### Organisms isolated from positive culture episodes

*S. aureus* was the most frequently isolated pathogen, occurring in 187 positive episode–organism records, followed by *E. coli* (*n* = 120) and *Klebsiella pneumoniae* (*n* = 97) (Table 2). Confirmed anaerobic-bottle contribution was observed in 59 of 187 *S. aureus* records (31.6%), 35 of 120 *E. coli* records (29.2%), and 23 of 66 records involving viridans group streptococci (34.8%). Contribution was less frequent for *K. pneumoniae* (11/97, 11.3%) and uncommon for *Pseudomonas aeruginosa* (1/37, 2.7%). Among 60 records involving *Staphylococcus epidermidis* that met the repeated-set criterion for classification as a pathogen, 7 (11.7%) showed confirmed contribution.

**TABLE 2:** Organism-specific episode-level diagnostic contribution of anaerobic bottles^*a*^

Organism	Positive records, *n*	Evaluable records, *n*	Confirmed contribution, *n* (%)	Additional recovery, *n*	Earlier detection, *n*	Indeterminate, *n*
*Staphylococcus aureus*	187	179	59 (31.6)	31	28	8
*Escherichia coli*	120	116	35 (29.2)	30	5	4
*Klebsiella pneumoniae*	97	94	11 (11.3)	11	0	3
Viridans group streptococci	66	55	23 (34.8)	22	1	11
*Staphylococcus epidermidis*	60	53	7 (11.7)	3	4	7
*Enterobacter cloacae* complex	46	42	3 (6.5)	3	0	4
*Pseudomonas aeruginosa*	37	32	1 (2.7)	1	0	5
*Enterococcus faecium*	32	29	5 (15.6)	5	0	3
*Enterococcus faecalis*	29	25	2 (6.9)	2	0	4
*Streptococcus agalactiae*	20	20	3 (15.0)	2	1	0
*Candida guilliermondii*	19	19	0 (0.0)	0	0	0
*Streptococcus pneumoniae*	19	19	3 (15.8)	3	0	0
*Candida krusei*	18	18	0 (0.0)	0	0	0
*Candida albicans*	17	16	0 (0.0)	0	0	1
*Serratia marcescens*	17	12	0 (0.0)	0	0	5
*Candida parapsilosis*	12	12	1 (8.3)	1	0	0
*Staphylococcus haemolyticus*	12	10	0 (0.0)	0	0	2
*Stenotrophomonas maltophilia*	12	12	0 (0.0)	0	0	0
*Burkholderia cepacia* complex	11	11	0 (0.0)	0	0	0
*Candida glabrata*	11	9	4 (36.4)	2	2	2
*Bacillus* spp. (non-*B*. *cereus* group)	10	10	2 (20.0)	2	0	0

^*a*^ Data are presented as episode–organism records; therefore, polymicrobial episodes could contribute more than one record. Only organisms identified in at least 10 positive episode–organism records are shown. Percentages for confirmed contribution were calculated using all positive episode–organism records for the corresponding organism as the denominator. Confirmed contribution comprised additional recovery or earlier detection, with contribution-evaluable and indeterminate records shown separately.

Obligate anaerobes were identified in 11 positive episodes, comprising 12 episode–organism records. At the episode level, nine showed additional recovery, one earlier detection, and one was indeterminate (Table S1). Thus, most anaerobic-bottle contribution was attributable to facultative organisms. A total of 54 positive episodes were polymicrobial; 12 showed confirmed contribution, and 30 were indeterminate.

### Diagnostic contribution according to sampling location

Anaerobic-bottle contribution varied across sampling locations (Table 3). The proportion of positive episodes with confirmed contribution was highest in the emergency room (109/387, 28.2%), followed by the NICU (28/112, 25.0%), general ward (43/250, 17.2%), PICU (15/89, 16.9%), and hemato-oncology ward (13/161, 8.1%). When all multi-set episodes were used as the denominator, the absolute added yield was highest in the NICU (15.1 per 1,000 episodes), followed by the PICU (10.1 per 1,000), emergency room (9.9 per 1,000), general ward (7.9 per 1,000), and hemato-oncology ward (2.8 per 1,000). Additional recovery accounted for most contribution at every location. Organism profiles also differed by sampling location. *E. coli* was the most frequently identified organism in the emergency room, whereas *S. aureus* was most frequent in the general ward, NICU, PICU, and hemato-oncology ward (Table S2).

**TABLE 3:** Anaerobic-bottle diagnostic contribution according to sampling location^*a*^^,^^*b*^

Sampling location	All multi-set episodes, *n*	Positive episodes, *n* (%)	Confirmed contribution, *n* (% of positive)	Additional recovery, *n*	Earlier detection, *n*	Indeterminate positive episodes, *n*	Absolute added yield per 1,000 episodes
General ward	5,472	250 (4.6)	43 (17.2)	34	9	14	7.9
Emergency room	11,039	387 (3.5)	109 (28.2)	90	19	8	9.9
NICU	1,855	112 (6.0)	28 (25.0)	16	12	5	15.1
PICU	1,487	89 (6.0)	15 (16.9)	9	6	6	10.1
Hemato-oncology	4,632	161 (3.5)	13 (8.1)	10	3	10	2.8
Total	24,485	999 (4.1)	208 (20.8)	159	49	43	8.5

^*a*^ NICU, neonatal intensive care unit; PICU, pediatric intensive care unit.

^*b*^ Percentages for positive episodes use all multi-set episodes at each location as the denominator. Percentages for confirmed contribution use all positive episodes at that location. Confirmed contribution comprised additional recovery or earlier detection. Absolute added yield was calculated per 1,000 multi-set episodes.

### Factors associated with anaerobic-bottle contribution

In multivariable logistic regression among 956 contribution-evaluable positive episodes, sampling location was associated with anaerobic-bottle contribution (global robust Wald *P* < 0.001), whereas age group, sex, and calendar year were not significant overall (Table 4). Compared with the general ward, the emergency room had higher adjusted odds of contribution (adjusted odds ratio [aOR], 1.83; 95% confidence interval [CI], 1.23–2.73), whereas the hemato-oncology ward had lower odds (aOR, 0.43; 95% CI, 0.21–0.85).

**TABLE 4:** Multivariable logistic regression for factors associated with anaerobic-bottle contribution^*a*^^*,^b^*^

Characteristic	Adjusted odds ratio	95% CI	*P*-value
Age 1–4 years vs 5–17 years	1.20	0.77–1.88	0.424
Age <1 year vs 5–17 years	1.05	0.66–1.66	0.852
Female vs male	0.78	0.54–1.11	0.162
Emergency room vs general ward	1.83	1.23–2.73	0.003
NICU vs general ward	1.70	0.78–3.68	0.180
PICU vs general ward	0.97	0.46–2.07	0.945
Hemato-oncology vs general ward	0.43	0.21–0.85	0.016

^*a*^ CI, confidence interval; NICU, neonatal intensive care unit; PICU, pediatric intensive care unit.

^*b*^ The outcome was confirmed anaerobic-bottle contribution among 956 contribution-evaluable positive episodes from 525 patients. Adjusted odds ratios were estimated using multivariable logistic regression with patient-clustered robust standard errors. The model included age group, sex, sampling location, and calendar year; calendar-year coefficients are not shown. Reference categories were age 5–17 years, male sex, general ward, and calendar year 2020. Global robust Wald *P*-values: age group, 0.722; sex, 0.162; sampling location, <0.001; calendar year, 0.213.

Collection source composition differed across locations. After additional adjustment for episode-level source composition, the global location association remained significant (*P* = 0.044); the emergency-room estimate remained elevated (aOR, 1.52; 95% CI, 1.02–2.28), whereas the hemato-oncology estimate was attenuated (aOR, 0.60; 95% CI, 0.30–1.21). Compared with peripheral-only episodes, line-only and mixed peripheral/line episodes had lower odds of contribution (aOR, 0.39 [95% CI, 0.20–0.75] and 0.43 [95% CI, 0.28–0.65], respectively). Adjustment for any CCC did not materially change the location estimates (Table S3).

Among children younger than 4 years, 96 of 441 positive episodes showed confirmed contribution (21.8%), corresponding to 8.8 per 1,000 multi-set episodes. In an outcome-independent sensitivity analysis restricted to the earliest multi-set episode per clinical encounter, the absolute added yield was similar (9.3 per 1,000 episodes), although the contribution proportion was higher (26.2%), and the location association was attenuated (Table S3).

### Potential contaminants and common-commensal recovery

Among 393 monomicrobial potential-contaminant paired sets, 157 were positive only in the anaerobic bottle, 140 only in Peds Plus/F, and 96 in both bottles; the discordant recovery difference was not significant (exact McNemar *P* = 0.353). Among 4,675 single-paired-set episodes, 105 (2.25%) had common-commensal-only recovery. These episodes were not adjudicated as contaminants because no independently accessioned companion set was available (Table S4).

### Within-accession and organism-specific TTP analyses

In the direct within-accession analysis of 2,615 paired sets with a final positive laboratory report, 892 were positive only in the Peds Plus/F bottle, 499 only in the anaerobic bottle, and 1,224 in both bottles (exact McNemar *P* < 0.001; Fig. 1B; Table S5). Among 1,156 both-positive monomicrobial paired sets, the median anaerobic-minus-Peds Plus/F TTP difference was −0.17 h (IQR, −1.33 to 1.17 hours), with no significant overall paired difference between bottle types (paired Wilcoxon *P* = 0.355; Table S6). In the subset of 1,012 paired sets yielding monomicrobial pathogens, the median difference was similarly small (−0.17 h [IQR, −1.36 to 1.00 h]), although the paired Wilcoxon test was statistically significant (*P* = 0.007).

**Fig 2:**
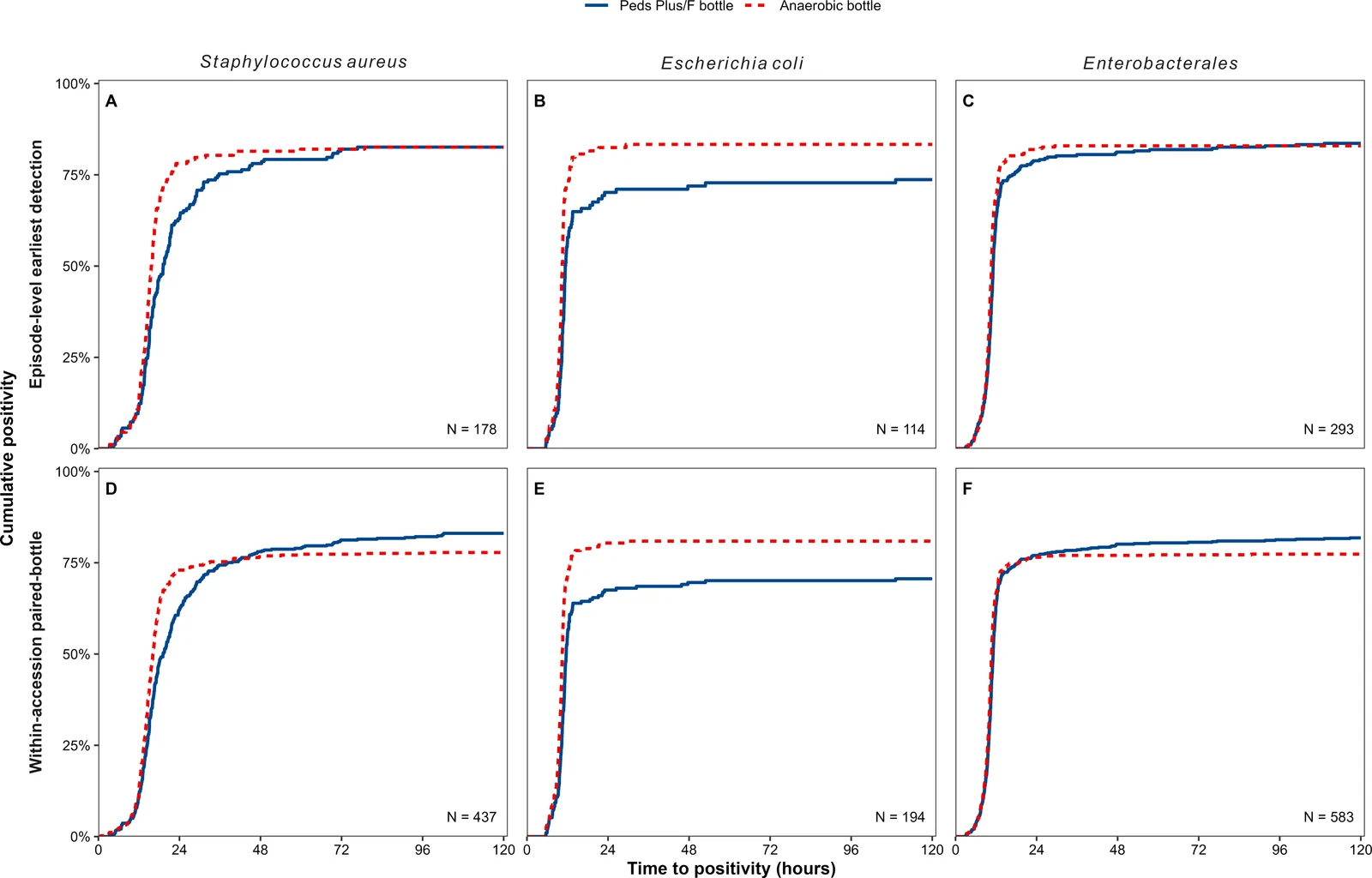
Kaplan–Meier cumulative positivity curves for *Staphylococcus aureus*, *Escherichia coli*, and *Enterobacterales*. Panels **A and D **show *S. aureus*, panels **B and E** show *E. coli*, and panels **C and F** show *Enterobacterales*. Panels **A–C** compare the earliest episode-level TTP by bottle type within receipt-defined multi-set episodes; panels **D–F** show within-accession comparisons restricted to monomicrobial paired sets. Target-organism nonrecovery by a bottle type was right-censored at 120 h. Curves are presented as one minus the Kaplan–Meier survival estimate; curve height at 120 h represents the proportion of target-positive units detected by each bottle type. N denotes episode–organism units in panels **A–C** and accession-defined paired sets in panels **D–F**. *Enterobacterales* includes *E. coli*. Statistical comparisons are reported in Table S7. TTP, time to positivity.

Cumulative-positivity curves showed organism-specific detection patterns at both the episode and within-accession levels (Fig. 2 and Table S7). For *S. aureus*, anaerobic bottles showed earlier detection at both levels, with median anaerobic-minus-Peds Plus/F differences of −1.59 h at the episode level and −1.26 h within accessions. Final recovery was identical between bottle types at the episode level and numerically higher in Peds Plus/F bottles within accessions (83.1% versus 77.8%; exact McNemar *P* = 0.092). For *E. coli*, anaerobic bottles showed earlier detection and numerically higher final recovery at both levels; within accessions, recovery was 80.9% versus 70.6% (unadjusted exact McNemar *P* = 0.049). For *Enterobacterales* overall, the earlier-detection difference was modest (median difference, −0.33 h at both levels), whereas final recovery was similar at the episode level and numerically higher in Peds Plus/F bottles within accessions (81.8% versus 77.4%; *P* = 0.105). Within accessions, an *Enterobacterales* lead exceeding 4 h occurred equally often in the two directions (14 paired sets each). Patient-clustered matched-strata Cox models, which incorporated both detection timing and exclusive recovery, favored anaerobic detection for all three organism groups at both analytic levels (all *P* ≤ 0.020). Exploratory Firth regression suggested lower odds of an episode-level anaerobic-bottle lead of more than 4 h among children aged <4 years than among those aged 4–17 years (OR, 0.45; 95% CI, 0.20–0.95; *P* = 0.035; Table S8).

## DISCUSSION

In this hospital-wide episode-level analysis, anaerobic bottles contributed to 20.8% of positive multi-set pediatric blood culture episodes. The contribution was driven largely by facultative pathogens, particularly *S. aureus*, *E. coli*, viridans group streptococci, and *S. epidermidis*, and occurred more often through additional organism recovery than through detection more than 4 h earlier. Obligate anaerobes were identified in only 11 positive episodes. Contribution was highest in the emergency room and NICU in descriptive analyses, and the emergency room association persisted after adjustment for collection source composition. Although the overall within-accession TTP difference was small, organism-specific analyses showed earlier anaerobic detection for selected organisms. These findings suggest that the main value of anaerobic bottles in pediatric practice lies in complementary recovery of clinically important facultative pathogens rather than in the uncommon detection of obligate anaerobes alone.

Earlier pediatric literature generally supported selective anaerobic bottle use because obligate anaerobic bacteremia is uncommon (9). More recent pediatric and paired-bottle studies, however, have shown improved recovery of clinically relevant pathogens and, in selected settings, shorter time to positivity, supporting anaerobic bottle use beyond obligate anaerobe detection alone (11–16). Our findings largely confirm this contemporary literature; the principal extension is a hospital-wide episode-level evaluation across multiple pediatric settings, with paired sets defined by laboratory accession and supported by direct within-accession analyses. Together, these findings frame anaerobic bottles as part of diagnostic stewardship rather than a tool limited to rare anaerobic bacteremia (8).

The organism-specific pattern is biologically plausible because facultative anaerobes can adapt to fluctuating oxygen conditions at infectious foci (23). Evidence is relatively direct for *S. aureus*, for which higher anaerobic-bottle recovery has been reported in both older FAN-bottle work and a modern paired-bottle study (15, 24). For *E. coli*, supportive evidence comes from *Enterobacterales* studies showing earlier anaerobic-bottle positivity and shorter detection times in BACTEC Lytic/10 Anaerobic/F bottles (25–27). Our results similarly showed earlier anaerobic detection for selected organisms, but final recovery varied by organism and was not uniformly greater in the anaerobic bottle.

These organism-specific differences should also be interpreted in light of the aerobic comparator used in this study, which was Peds Plus/F rather than a conventional adult aerobic bottle. Peds Plus/F is intended for lower-volume specimens and differs from Plus Aerobic/F in its recommended inoculum and medium characteristics, whereas Lytic/10 Anaerobic/F differs from Peds Plus/F in formulation as well as incubation atmosphere (28). Experimental comparisons have shown that Peds Plus/F and standard aerobic media may differ in organism recovery and time to positivity under low-volume conditions, although the magnitude of these differences varies by organism and system (28, 29). Accordingly, the observed differences reflect the pragmatic performance of the routine Peds Plus/F–Lytic/10 Anaerobic/F pair and should not be interpreted as isolating the effect of anaerobic incubation alone. The more frequent Peds Plus/F-only recovery in the direct within-accession analysis further supports a complementary, rather than hierarchical, relationship between the two bottle types.

The location-specific distribution of anaerobic bottle contribution also has direct practical implications. The relatively high contribution observed in the NICU and emergency room suggests that paired aerobic–anaerobic collection may be particularly useful in settings where early microbiologic clarification can influence initial antimicrobial decisions and where repeat sampling may be difficult (1, 8). The NICU pattern is biologically plausible because *E. coli* is a leading early-onset neonatal pathogen, particularly among preterm infants, whereas *S. aureus* remains an important late-onset, often device-associated pathogen; both showed substantial anaerobic-bottle contribution in our cohort (30–33). This interpretation is consistent with prior pediatric, neonatal, and intensive care studies showing additional recovery and, in selected subsets, earlier positivity with anaerobic bottles, although the magnitude of benefit may vary with local epidemiology and patient mix (11–14, 16). These between-location differences may partly reflect variation in organism profiles and collection-source composition. The hemato-oncology ward had a distinct organism distribution, with *Candida guilliermondii* and *P. aeruginosa* among its five most frequently identified organisms (Table S2). These organisms showed no or minimal confirmed anaerobic-bottle contribution in the overall cohort (0/19 and 1/37 [2.7%], respectively; Table 2). The lower contribution observed in the hemato-oncology ward may therefore reflect both its organism distribution and collection-source composition, as the corresponding estimate was attenuated after source adjustment, whereas the emergency-room association persisted. Adjustment for CCC-defined underlying medical complexity did not materially change the location estimates.

These findings should be interpreted alongside the central importance of blood volume in pediatric blood culture practice. Because bacteremia detection is volume dependent and inadequate submitted volume is common in children, the practical priority is to obtain the recommended weight-based total volume and allocate it appropriately between bottles (2–5). Our institutional protocol specified approximately equal division of the volume assigned to each paired set between the Peds Plus/F and anaerobic bottles, even when only a small total volume was available. However, actual bottle-level inoculated volumes were unavailable, so adherence to this strategy could not be verified, and our data cannot define a minimum-volume threshold below which an aerobic-only strategy would be preferable. Neonatal and pediatric intervention studies suggest that paired-bottle allocation and volume standardization can improve pathogen recovery (10, 17, 18), supporting their integration into broader blood culture stewardship efforts (8). Although the overall contribution proportion was similar among children younger than 4 years, exploratory Firth analysis suggested lower odds of an anaerobic-bottle lead of more than 4 h among both-positive episode–organism units in this age group. Because this analysis assessed earlier detection only and excluded additional organism recovery, it should not be interpreted as evidence of lower overall anaerobic-bottle contribution in younger children.

A major strength of this study is its hospital-wide episode-level design, which grouped contemporaneously submitted accession-defined paired sets into 15-min receipt-defined episodes and included all eligible multi-set episodes. Repeated observations within patients were accounted for in the multivariable regression and Cox models. This design complements prior bottle-level or unit-specific studies by estimating anaerobic-bottle contribution during the contemporaneous culture episode across routine pediatric practice (11–16). Other strengths include direct within-accession paired-bottle comparisons, inclusion of polymicrobial episodes, organism- and location-specific analyses, and assessment of both proportional contribution and absolute added yield.

Several limitations should be acknowledged. This retrospective single-center study used one blood culture platform and a specific Peds Plus/F–Lytic/10 Anaerobic/F bottle pair, which may limit generalizability to other systems and collection practices. Bottle-level inoculated volumes were unavailable; therefore, we could not stratify analyses by total volume or derive minimum-volume or allocation thresholds, despite the established influence of volume on pediatric blood culture performance (2–5, 10). Nevertheless, the age-based subgroup analysis does not replace measured-volume analysis or exclude unequal inoculation between bottles. Because the effects of inoculum volume, bottle formulation, and incubation atmosphere could not be separated, the within-accession results reflect the pragmatic performance of the routine bottle pair rather than the intrinsic superiority of either bottle. Actual venipuncture times were unavailable, and the 15-min receipt window served as an operational proxy; however, direct within-accession analyses were independent of this window. Single-paired-set episodes were excluded from the primary analysis because common-commensal recovery could not be adjudicated across independently accessioned sets, and potential contaminants were classified using a CLSI M47-informed laboratory algorithm without clinical adjudication (21). Finally, bottle attribution was incomplete in some polymicrobial episodes, and effects on antimicrobial management or clinical outcomes were not assessed.

Anaerobic bottles provided complementary diagnostic information in approximately one-fifth of positive pediatric multi-set blood culture episodes, mainly through additional recovery of facultative pathogens rather than detection of obligate anaerobes or uniformly earlier positivity. These findings largely support contemporary pediatric studies while extending them through a hospital-wide episode-level analysis across diverse clinical settings. Because direct within-accession recovery favored Peds Plus/F overall, the results support complementary paired use when adequate blood volume is available, rather than indicating intrinsic superiority of the anaerobic bottle.

## Data Availability

The individual-level data used in this study are not publicly available because they contain potentially identifiable patient information and are subject to institutional and IRB restrictions. De-identified data necessary to reproduce the analyses and the analysis code may be made available upon reasonable request, subject to institutional approval and applicable data-use agreements.

## SUPPLEMENTAL MATERIAL

Tables S1 to S8 jcm.00764-26-s0001.docx Obligate anaerobe detection patterns, location-specific organism distributions, sensitivity analyses, potential-contaminant and single-paired-set findings, within-accession recovery and TTP comparisons, organism-specific analyses, and exploratory Firth regression.

ASM does not own the copyrights to Supplemental Material that may be linked to, or accessed through, an article. The authors have granted ASM a non-exclusive, world-wide license to publish the Supplemental Material files. Please contact the corresponding author directly for reuse.
