# Supplementary material for: Diagnostic contribution of anaerobic bottles in pediatric blood cultures: experience at a tertiary children’s hospital
Source: J Clin Microbiol. 2026 Sep 30;64(11):e00764-26. doi: 10.1128/jcm.00764-26 (PMC13753796; doi:10.1128/jcm.00764-26)
Supplement: Supplementary file 1 [file jcm.00764-26-s0001.docx]

**Supplementary Table S1.** Obligate anaerobes identified in positive multi-set episodes and their bottle-specific detection patterns

| **Episode No.** | **Organism** | **Sampling location** | **Recovered from anaerobic bottle** | **Recovered from Peds Plus/F bottle** | **Earliest anaerobic TTP, h** | **Earliest Peds Plus/F TTP, h** | **Anaerobic-bottle contribution** |
| --- | --- | --- | --- | --- | --- | --- | --- |
| 1 | *Anaerococcus* spp. | Emergency room | Yes | No | 50.07 | — | Additional recovery |
| 2 | *Bacteroides fragilis* | Emergency room | Yes | No | 21.14 | — | Additional recovery |
| 3 | *Bacteroides thetaiotaomicron* | Emergency room | Yes | No | 26.12 | — | Additional recovery |
| 4 | *Alistipes onderdonkii* | Emergency room | Yes | No | Not attributable | — | Additional recovery |
| 4 | *Eggerthella lenta* | Emergency room | Yes | No | Not attributable | — | Additional recovery |
| 5 | *Finegoldia* spp. | Emergency room | Yes | No | 36.27 | — | Additional recovery |
| 6 | *Fusobacterium* spp. | Emergency room | Yes | No | 19.09 | — | Additional recovery |
| 7 | *Fusobacterium* spp. | Hemato-oncology | Yes | Yes | 24.19 | 39.24 | Earlier detection |
| 8 | *Veillonella parvula* | Emergency room | Not attributable | Not attributable | Not attributable | Not attributable | Indeterminate* |
| 9 | *Veillonella* spp. | Emergency room | Yes | No | 46.55 | — | Additional recovery |
| 10 | *Veillonella* spp. | Hemato-oncology | Yes | No | 26.55 | — | Additional recovery |
| 11 | *Veillonella* spp. | General ward | Yes | No | 27.15 | — | Additional recovery |

Abbreviations: TTP, time to positivity.

An episode could contribute more than one organism record. Episode 4 contained two obligate anaerobes. In polymicrobial paired sets, bottle-specific recovery could be assigned when only one bottle type was positive, but organism-specific TTP was not attributed to individual organisms.

*One paired set in this episode yielded *Pseudomonas aeruginosa* and *V. parvula*, whereas the other yielded *P. aeruginosa* alone. Because *V. parvula* in the polymicrobial paired set could not be assigned specifically to the Peds Plus/F or anaerobic bottle, bottle-specific recovery and TTP were not attributable; its anaerobic-bottle contribution was therefore classified as indeterminate.

**Supplementary Table S2.** Most frequently identified organisms according to sampling location

| **Sampling location** | **Rank** | **Organism** | **Positive episodes with organism, n (%)** | **Positive episodes at location, n** |
| --- | --- | --- | --- | --- |
| General ward | 1 | *Staphylococcus aureus* | 64 (25.6) | 250 |
| General ward | 2 | *Staphylococcus epidermidis* | 22 (8.8) | 250 |
| General ward | 3 | *Klebsiella pneumoniae* | 17 (6.8) | 250 |
| General ward | 4 | *Escherichia coli* | 16 (6.4) | 250 |
| General ward | 5 | Viridans group streptococci | 15 (6.0) | 250 |
| Emergency room | 1 | *Escherichia coli* | 82 (21.2) | 387 |
| Emergency room | 2 | *Klebsiella pneumoniae* | 52 (13.4) | 387 |
| Emergency room | 3 | *Staphylococcus aureus* | 47 (12.1) | 387 |
| Emergency room | 4 | Viridans group streptococci | 39 (10.1) | 387 |
| Emergency room | 5 | *Enterobacter cloacae* complex | 22 (5.7) | 387 |
| NICU | 1 | *Staphylococcus aureus* | 28 (25.0) | 112 |
| NICU | 2 | *Escherichia coli* | 15 (13.4) | 112 |
| NICU | 3 | *Staphylococcus epidermidis* | 13 (11.6) | 112 |
| NICU | 4 | *Candida albicans* | 8 (7.1) | 112 |
| NICU | 5 | *Klebsiella pneumoniae* | 7 (6.2) | 112 |
| NICU | 5 | *Serratia marcescens* | 7 (6.2) | 112 |
| PICU | 1 | *Staphylococcus aureus* | 26 (29.2) | 89 |
| PICU | 2 | *Staphylococcus epidermidis* | 11 (12.4) | 89 |
| PICU | 3 | *Candida albicans* | 7 (7.9) | 89 |
| PICU | 3 | *Enterococcus faecalis* | 7 (7.9) | 89 |
| PICU | 3 | *Klebsiella pneumoniae* | 7 (7.9) | 89 |
| Hemato-oncology | 1 | *Staphylococcus aureus* | 22 (13.7) | 161 |
| Hemato-oncology | 2 | *Candida guilliermondii* | 17 (10.6) | 161 |
| Hemato-oncology | 3 | *Enterobacter cloacae* complex | 14 (8.7) | 161 |
| Hemato-oncology | 3 | *Klebsiella pneumoniae* | 14 (8.7) | 161 |
| Hemato-oncology | 5 | *Pseudomonas aeruginosa* | 13 (8.1) | 161 |

Abbreviations: NICU, neonatal intensive care unit; PICU, pediatric intensive care unit.

Organisms tied at the cutoff rank were included. Because polymicrobial episodes could contribute more than one organism, percentages within a location may sum to more than 100%.

**Supplementary Table S3.** Sensitivity analyses with additional adjustment for collection-source composition or pediatric complex chronic conditions and restriction to the earliest episode per clinical encounter

| **Sensitivity analysis** | **Characteristic** | **aOR (95% CI)** | ***P* value** | **Global *P* for sampling location** |
| --- | --- | --- | --- | --- |
| Collection-source adjusted model | Emergency room vs general ward | 1.52 (1.02–2.28) | 0.041 | 0.044 |
| Collection-source adjusted model | NICU vs general ward | 1.34 (0.62–2.94) | 0.458 | 0.044 |
| Collection-source adjusted model | PICU vs general ward | 1.30 (0.59–2.86) | 0.517 | 0.044 |
| Collection-source adjusted model | Hemato-oncology vs general ward | 0.60 (0.30–1.21) | 0.157 | 0.044 |
| Collection-source adjusted model | Line only vs peripheral only | 0.39 (0.20–0.75) | 0.005 |  |
| Collection-source adjusted model | Mixed peripheral/line vs peripheral only | 0.43 (0.28–0.65) | <0.001 |  |
| Any-CCC adjusted model | Emergency room vs general ward | 1.71 (1.14–2.56) | 0.009 | <0.001 |
| Any-CCC adjusted model | NICU vs general ward | 1.96 (0.89–4.30) | 0.094 | <0.001 |
| Any-CCC adjusted model | PICU vs general ward | 1.04 (0.50–2.18) | 0.909 | <0.001 |
| Any-CCC adjusted model | Hemato-oncology vs general ward | 0.47 (0.24–0.95) | 0.036 | <0.001 |
| Any-CCC adjusted model | Any CCC vs no CCC | 0.52 (0.34–0.81) | 0.004 |  |
| Earliest multi-set episode per clinical encounter | Emergency room vs general ward | 1.70 (1.03–2.78) | 0.037 | 0.140 |
| Earliest multi-set episode per clinical encounter | NICU vs general ward | 2.13 (0.81–5.61) | 0.126 | 0.140 |
| Earliest multi-set episode per clinical encounter | PICU vs general ward | 2.52 (0.85–7.46) | 0.096 | 0.140 |
| Earliest multi-set episode per clinical encounter | Hemato-oncology vs general ward | 0.92 (0.36–2.36) | 0.859 | 0.140 |

Abbreviations: aOR, adjusted odds ratio; CI, confidence interval; CCC, pediatric complex chronic conditions; NICU, neonatal intensive care unit; PICU, pediatric intensive care unit.

All logistic regression models were restricted to contribution-evaluable positive episodes and used patient-clustered robust standard errors. The collection-source- and CCC-adjusted models included age group, sex, sampling location, calendar year, and episode-level source composition or any CCC, respectively; the global *P* value for source composition was <0.001. The encounter-level sensitivity model included age group, sex, sampling location, and calendar year and was restricted to the earliest eligible multi-set episode per clinical encounter. For context, the corresponding encounter-level cohort comprised 16,431 episodes, including 580 positive episodes, of which 152 showed confirmed anaerobic-bottle contribution (26.2%; 9.3 per 1,000 episodes).

**Supplementary Table S4.** Bottle-recovery patterns of potential contaminants and common-commensal recovery in single-paired-set episodes

| **Cohort/analysis** | **Measure** | **Count** | **Denominator** | **Percent or *P* value** |
| --- | --- | --- | --- | --- |
| Primary multi-set cohort: potential-contaminant paired-set-organism records | Anaerobic only | 162 | 416 | 38.9% |
| Primary multi-set cohort: potential-contaminant paired-set-organism records | Growth in both bottles | 110 | 416 | 26.4% |
| Primary multi-set cohort: potential-contaminant paired-set-organism records | Peds Plus/F only | 144 | 416 | 34.6% |
| Monomicrobial potential-contaminant paired sets | Anaerobic only | 157 | 393 | 39.9% |
| Monomicrobial potential-contaminant paired sets | Peds Plus/F only | 140 | 393 | 35.6% |
| Monomicrobial potential-contaminant paired sets | Growth in both bottles | 96 | 393 | 24.4% |
| Monomicrobial potential-contaminant paired sets | Exact McNemar test |  |  | 0.353 |
| Single-paired-set episodes | Any common-commensal recovery (not adjudicable) | 112 | 4,675 | 2.40% |
| Single-paired-set episodes | Common-commensal-only recovery (not adjudicable) | 105 | 4,675 | 2.25% |
| Single-paired-set episodes | Episodes with a noncommensal pathogen | 218 | 4,675 | 4.66% |
| Single-paired-set episodes | Negative episodes | 4,352 | 4,675 | 93.09% |

Potential contaminants were defined only within multi-set episodes. In single-paired-set episodes, common-commensal recovery was reported descriptively and was not adjudicated as contamination because no independently accessioned companion set was available. The categories “any common-commensal recovery” and “presence of a noncommensal pathogen” were not mutually exclusive; 7 episodes met both criteria. By contrast, common-commensal-only recovery, presence of a noncommensal pathogen, and negative episodes were mutually exclusive categories and together accounted for all 4,675 single-paired-set episodes.

**Supplementary Table S5.** Direct within-accession paired-bottle recovery patterns

| **Analysis** | **Paired sets, n** | **Anaerobic only, n (%)** | **Peds Plus/F only, n (%)** | **Growth in both bottles, n (%)** | **Anaerobic recovery, n (%)** | **Peds Plus/F recovery, n (%)** | **Matched OR (95% CI)** | **Exact McNemar *P* value** |
| --- | --- | --- | --- | --- | --- | --- | --- | --- |
| All paired sets with a final positive laboratory report | 2615 | 499 (19.1%) | 892 (34.1%) | 1224 (46.8%) | 1723 (65.9%) | 2116 (80.9%) | 0.56 (0.50–0.62) | <0.001 |
| Monomicrobial pathogens | 2028 | 307 (15.1%) | 704 (34.7%) | 1017 (50.1%) | 1324 (65.3%) | 1721 (84.9%) | 0.44 (0.38–0.50) | <0.001 |

Abbreviations: OR, odds ratio; CI, confidence interval.

Matched odds ratios compare anaerobic-only versus Peds Plus/F-only discordant paired sets.

**Supplementary Table S6.** Overall paired time-to-positivity summaries among both-positive monomicrobial paired sets

| **Analysis** | **Paired sets, n** | **Patients, n** | **Anaerobic TTP, median [IQR], h** | **Peds Plus/F TTP, median [IQR], h** | **Anaerobic minus Peds Plus/F TTP, median [IQR], h** | **Anaerobic >4 h earlier, n** | **Within 4 h, n** | **Peds Plus/F >4 h earlier, n** | **Paired Wilcoxon P** |
| --- | --- | --- | --- | --- | --- | --- | --- | --- | --- |
| All both-positive monomicrobial paired sets | 1156 | 399 | 13.09 [9.89–20.10] | 13.14 [10.05–20.12] | -0.17 [-1.33–1.17] | 131 | 854 | 171 | 0.355 |
| Both-positive paired sets with a monomicrobial pathogen | 1012 | 321 | 12.33 [9.75–17.29] | 12.42 [9.80–17.79] | -0.17 [-1.36–1.00] | 119 | 762 | 131 | 0.007 |

Abbreviations: TTP, time to positivity; IQR, interquartile range.

TTP difference was calculated as anaerobic bottle TTP minus Peds Plus/F bottle TTP; negative values indicate earlier positivity in the anaerobic bottle.

**Supplementary Table S7.** Organism-specific episode-level and within-accession recovery and time-to-positivity analyses

| **Analysis level** | **Organism/group** | **Units, n** | **Detected, anaerobic / Peds Plus/F, n (%)** | **Exclusive recovery, anaerobic / Peds Plus/F, n;  McNemar *P*** | **Anaerobic − Peds Plus/F TTP among both-positive units, median [IQR], h; n; Wilcoxon *P*** | **>4-h lead, anaerobic / Peds Plus/F, n** | **Clustered Cox HR (95% CI); *P*** |
| --- | --- | --- | --- | --- | --- | --- | --- |
| Episode level | *Staphylococcus aureus* | 178 | 147 (82.6%) /  147 (82.6%) | 31 / 31; *P* = 1.000 | −1.59 [−4.00 to 0.05]; n = 116; *P* < 0.001 | 28 / 8 | 1.87 (1.28–2.73); *P* = 0.001 |
| Episode level | *Escherichia coli* | 114 | 95 (83.3%) /   84 (73.7%) | 30 / 19; *P* = 0.152 | −0.67 [−1.50 to −0.33]; n = 65; *P* < 0.001 | 5 / 2 | 3.07 (1.95–4.83); *P* < 0.001 |
| Episode level | *Enterobacterales* | 293 | 243 (82.9%) /  245 (83.6%) | 48 / 50; P = 0.920 | −0.33 [−0.84 to 0.09]; n = 195; *P* < 0.001 | 8 / 9 | 1.82 (1.40–2.35); *P* < 0.001 |
| Within accession | *Staphylococcus aureus* | 437 | 340 (77.8%) /  363 (83.1%) | 74 / 97; *P* = 0.092 | −1.26 [−3.83 to 0.33]; n = 266; *P* < 0.001 | 63 / 18 | 1.51 (1.07–2.14); *P* = 0.020 |
| Within accession | *Escherichia coli* | 194 | 157 (80.9%) /  137 (70.6%) | 57 / 37; *P* = 0.049 | −0.67 [−1.50 to −0.33]; n = 100; *P* < 0.001 | 7 / 4 | 2.96 (2.09–4.19); *P* < 0.001 |
| Within accession | *Enterobacterales* | 583 | 451 (77.4%) /  477 (81.8%) | 106 / 132; *P* = 0.105 | −0.33 [−0.83 to 0.14]; n = 345; *P* < 0.001 | 14 / 14 | 1.50 (1.24–1.82); *P* < 0.001 |

Abbreviations: TTP, time to positivity; IQR, interquartile range; HR, hazard ratio; CI, confidence interval.

Episode-level analyses compared the earliest target-organism TTP by bottle type within each receipt-defined multi-set episode, requiring attributable bottle-specific recovery and valid organism-attributable TTPs for all positive bottle types. Within-accession analyses directly compared the two bottles sharing one accession and were restricted to monomicrobial paired sets. Detection percentages and McNemar tests included all eligible target-positive units. TTP differences, directional >4-hour counts, and paired Wilcoxon tests were restricted to both-positive units. Cox models included all eligible target-positive units, with nonrecovery from a bottle type right-censored at 120 hours and patient-clustered robust standard errors. Hazard ratios greater than 1 indicate an anaerobic-bottle detection advantage. TTP difference was calculated as anaerobic minus Peds Plus/F TTP; negative values indicate earlier anaerobic positivity. *Enterobacterales* includes *Escherichia coli*.

**Supplementary Table S8.** Exploratory Firth-penalized logistic regression for factors associated with an episode-level anaerobic-bottle lead of more than 4 hours

| **Characteristic** | **Odds ratio (95% CI)** | ***P* value** |
| --- | --- | --- |
| Age <4 years vs 4–17 years | 0.45 (0.20–0.95) | 0.035 |
| Emergency room vs general ward | 2.29 (0.97–5.94) | 0.059 |
| NICU vs general ward | 6.19 (2.00–20.67) | 0.002 |
| PICU vs general ward | 4.53 (1.38–14.73) | 0.014 |
| Hemato-oncology vs general ward | 0.76 (0.14–3.00) | 0.711 |
| Gram-positive cocci vs *Enterobacterales* and other Gram-negative rods | 3.36 (1.58–7.95) | 0.001 |
| Yeasts vs *Enterobacterales* and other Gram-negative rods | 5.86 (0.86–32.61) | 0.069 |
| Others vs *Enterobacterales* and other Gram-negative rods | 2.00 (0.20–10.41) | 0.495 |

Abbreviations: CI, confidence interval; NICU, neonatal intensive care unit; PICU, pediatric intensive care unit.

Firth penalized logistic regression included 492 episode–organism units from 303 patients, restricted to monomicrobial episodes with recovery from both bottle types and valid organism-attributable TTPs; 45 units (9.1%) had an anaerobic-bottle lead of more than 4 hours. Four of the 49 earlier-detection episodes were excluded as polymicrobial. The model simultaneously included age group, sampling location, and organism group. Reference categories were age 4–17 years and the general ward; the reference organism group comprised *Enterobacterales* and other Gram-negative rods. Odds ratios greater than 1 indicate higher odds of an anaerobic-bottle lead of more than 4 hours.
